# Ursodeoxycholic acid versus placebo in women with intrahepatic cholestasis of pregnancy (PITCHES): a randomised controlled trial

**DOI:** 10.1016/S0140-6736(19)31270-X

**Published:** 2019-09-07

**Authors:** Lucy C Chappell, Jennifer L Bell, Anne Smith, Louise Linsell, Edmund Juszczak, Peter H Dixon, Jenny Chambers, Rachael Hunter, Jon Dorling, Catherine Williamson, Jim G Thornton, PITCHES study group, Irshad Ahmed, Rita Arya, Virginia Beckett, Amarnath Bhide, Heather Brown, George Bugg, Helen Cameron, Nishigandh Deole, Madhuchanda Dey, James Dwyer, Leila Fahel, Ruta Gada, Joanna Girling, Anna Haestier, Sean Hughes, Radha Indusekhar, Bryony Jones, Rahila Khan, Alison Kirkpatrick, Ellen Knox, Karen Lincoln, Malcolm MacDougall, Franz Majoko, Karen McIntyre, Muna Noori, Wendy Oakley, Jane Preston, Poornima Ranka, Mumtaz Rashid, Marwan Salloum, Manjula Samyraju, Catharina Schram, Seema Sen, Sophia Stone, Bee Tan

**Affiliations:** a Department of Women and Children's Health, School of Life Course Sciences, King's College London, London, UK; b National Perinatal Epidemiology Unit Clinical Trials Unit, Nuffield Department of Population Health, University of Oxford, Oxford, UK; c ICP Support, Sutton Coldfield, UK; d Research Department of Primary Care and Population Health, University College London, London, UK; e Division of Neonatal-Perinatal Medicine, IWK Health Centre, Halifax, NS, Canada; f Division of Child Health, Obstetrics and Gynaecology, University of Nottingham, Nottingham, UK

## Abstract

**Background:**

Intrahepatic cholestasis of pregnancy, characterised by maternal pruritus and increased serum bile acid concentrations, is associated with increased rates of stillbirth, preterm birth, and neonatal unit admission. Ursodeoxycholic acid is widely used as a treatment without an adequate evidence base. We aimed to evaluate whether ursodeoxycholic acid reduces adverse perinatal outcomes in women with intrahepatic cholestasis of pregnancy.

**Methods:**

We did a double-blind, multicentre, randomised placebo-controlled trial at 33 hospital maternity units in England and Wales. We recruited women with intrahepatic cholestasis of pregnancy, who were aged 18 years or older and with a gestational age between 20 weeks and 40 weeks and 6 days, with a singleton or twin pregnancy and no known lethal fetal anomaly. Participants were randomly assigned 1:1 to ursodeoxycholic acid or placebo, given as two oral tablets a day at an equivalent dose of 500 mg twice a day. The dose could be increased or decreased at the clinician's discretion, to a maximum of four tablets and a minimum of one tablet a day. We recommended that treatment should be continued from enrolment until the infant's birth. The primary outcome was a composite of perinatal death (in-utero fetal death after randomisation or known neonatal death up to 7 days after birth), preterm delivery (<37 weeks' gestation), or neonatal unit admission for at least 4 h (from birth until hospital discharge). Each infant was counted once within this composite. All analyses were done according to the intention-to-treat principle. The trial was prospectively registered with the ISRCTN registry, number 91918806.

**Findings:**

Between Dec 23, 2015, and Aug 7, 2018, 605 women were enrolled and randomly allocated to receive ursodeoxycholic acid (n=305) or placebo (n=300). The primary outcome analysis included 304 women and 322 infants in the ursodeoxycholic acid group, and 300 women and 318 infants in the placebo group (consent to use data was withdrawn for 1 woman and 2 infants). The primary composite outcome occurred in 74 (23%) of 322 infants in the ursodeoxycholic acid group and 85 (27%) of 318 infants in the placebo group (adjusted risk ratio 0·85 [95% CI 0·62–1·15]). Two serious adverse events were reported in the ursodeoxycholic acid group and six serious adverse events were reported in the placebo group; no serious adverse events were regarded as being related to treatment.

**Interpretation:**

Treatment with ursodeoxycholic acid does not reduce adverse perinatal outcomes in women with intrahepatic cholestasis of pregnancy. Therefore, its routine use for this condition should be reconsidered.

**Funding:**

National Institute for Health Research Efficacy and Mechanism Evaluation Programme.

## Introduction

Intrahepatic cholestasis of pregnancy, also called obstetric cholestasis, is the most common liver disorder specific to pregnancy. The disease is characterised by maternal pruritus and increased serum bile acid concentrations, with maternal symptoms and abnormal biochemical test results typically resolving post partum. A systematic review and individual patient data meta-analysis^1^ from 2019 showed that intrahepatic cholestasis of pregnancy is associated with increased rates of spontaneous and iatrogenic preterm birth, meconium-stained amniotic fluid, and neonatal unit admission. In addition, the risk of stillbirth was found to be increased only for women with peak serum bile acid concentrations of 100 μmol/L or higher,^1^ in contrast to the previously held belief that the risk of stillbirth was increased for all women with intrahepatic cholestasis of pregnancy.^2^

Ursodeoxycholic acid, which is used outside of pregnancy to treat primary biliary cholangitis and other hepatobiliary disorders, has been used to treat intrahepatic cholestasis of pregnancy.^3^ Ursodeoxycholic acid is a naturally occurring bile acid that is present in small amounts in humans. It has several actions that result in the improvement of cholestasis, including increasing biliary bile acid excretion through upregulation of hepatic metabolising enzymes and bile acid transporters, stimulation of impaired hepatocellular secretion by post-transcriptional mechanisms, stabilisation of the plasma membrane and protection of cholangiocytes of the biliary epithelium against cytotoxicity of bile acids, and hepatocyte protection against bile acid-induced apoptosis.^4^, ^5^ Ursodeoxycholic acid is recommended in six national guidelines for management of intrahepatic cholestasis of pregnancy,^6^ principally for the improvement of maternal symptoms and biochemical test results. Surveys of practice have found that 97% of obstetricians in the UK use ursodeoxycholic acid for treating intrahepatic cholestasis of pregnancy.^7^

**Research in context**

**Evidence before this study**

The Cochrane systematic review on this topic, updated on Feb 20, 2013, concluded that “fewer instances of fetal distress/asphyxial events were seen in the ursodeoxycholic acid groups when compared with placebo but the difference was not statistically significant” and larger trials were needed.

**Added value of this study**

This trial is five times larger than the largest previous trial and nearly three times larger than all previous trials combined. Women were managed in a high-income health-care setting, with free access to care and regular surveillance (including repeated bile acid measurements), such that the trial is likely to represent current management of this condition.

**Implications of all the available evidence**

An updated systematic review and meta-analysis, including this trial, with the search conducted by the Cochrane Pregnancy and Childbirth Group (updated Dec 5, 2018), found that ursodeoxycholic acid does not reduce the incidence of stillbirth (incidence of one in the ursodeoxycholic acid group *vs* four in the placebo group, relative risk [RR] 0·39 [95% CI 0·08–1·93]; five trials, 891 participants; quality of evidence low) or spontaneous preterm birth before 37 weeks' gestation (RR 0·78 [95% CI 0·49–1·23]; three trials, 749 participants; quality of evidence high). However, ursodeoxycholic acid might reduce total (spontaneous and iatrogenic) preterm birth (RR 0·68 [95% CI 0·52–0·89]; three trials, 819 participants, including one low quality). Ursodeoxycholic acid does not reduce neonatal unit admission (RR 0·77 [95% CI 0·55–1·08]; two trials, 764 participants; quality of evidence high). Ursodeoxycholic acid does not seem to have any significant clinical benefit when used routinely for treatment of women with intrahepatic cholestasis of pregnancy.

Despite widespread recommendations of ursodeoxycholic acid in the treatment of intrahepatic cholestasis of pregnancy, the evidence base for its use is scant. Two meta-analyses had been done shortly before the inception of our trial.^8^, ^9^ One meta-analysis found that ursodeoxycholic acid was effective in reducing pruritus and improving liver test results in women with intrahepatic cholestasis of pregnancy, and might improve fetal outcomes; however, the largest randomised controlled trial included had only 84 participants.^8^ A subsequent Cochrane systematic review of the effectiveness of ursodeoxycholic acid for intrahepatic cholestasis of pregnancy concluded that it might ameliorate pruritus by a small amount, but that definitive evidence for improvement in perinatal outcomes was absent and large trials of ursodeoxycholic acid were needed to determine fetal benefits or risks.^9^ The Cochrane review found that many of the trials were at a moderate-to-high risk of bias, and the largest trial included only 111 women.^10^

We did a randomised placebo-controlled trial to evaluate whether ursodeoxycholic acid reduces adverse perinatal outcomes in women with intrahepatic cholestasis of pregnancy, and to investigate the effect of ursodeoxycholic acid on other short-term maternal and infant outcomes, and on the use of health-care resources.

## Methods

### Study design

We did a parallel-group, double-blind, multicentre, randomised placebo-controlled trial with individual randomisation to ursodeoxycholic acid or placebo using a 1:1 allocation ratio. The trial was done at 33 hospital maternity units in England and Wales. The trial was approved by the East of England—Essex Research Ethics Committee (15/EE/0010). The study protocol has been published previously.^11^ No substantial changes were made to the study design or methods after commencement of the trial.

### Participants

Women were eligible if the attending clinician considered that they had a diagnosis of intrahepatic cholestasis of pregnancy (defined as maternal pruritus with an increase in serum bile acid concentration above the upper limit of normal at randomly timed assessment, as measured in the local laboratory),^12^ and they were between 20 weeks and 40 weeks and 6 days of pregnancy on the day of randomisation, with a singleton or twin pregnancy, no known lethal fetal anomaly, aged 18 years or older, and able to give written informed consent. The diagnostic criteria were based on the UK standard-of-care guideline from the Royal College of Obstetricians and Gynaecologists, which defines intrahepatic cholestasis of pregnancy as “when otherwise unexplained pruritus occurs in pregnancy and abnormal liver function tests and/or raised bile acids occur in the pregnant woman and both resolve after delivery”.^12^ However, we required the more stringent criterion that all women had raised bile acid concentrations. In accordance with the UK guideline, all participating units used randomly timed bile acid concentrations for diagnostic and management purposes. Because we were recruiting prospectively to the trial, we could not confirm post-partum resolution at the time of enrolment. Women were not included in the trial if a decision had already been made for delivery within the next 48 h, if they had any known allergy to any component of the ursodeoxycholic acid or placebo tablets, or if they had a triplet or higher-order multiple pregnancy. 17 of the 33 maternity units used a threshold of 14 μmol/L as the upper limit of normal, whereas the remaining units used thresholds between 10 μmol/L and 13 μmol/L, according to local laboratory reference ranges. Research teams at each site approached women to confirm eligibility and provided verbal and written information. A trained clinician obtained written informed consent.

### Randomisation and masking

Participants were randomly allocated to receive ursodeoxycholic acid or placebo. Randomisation was done using a probabilistic minimisation algorithm to ensure approximate balance within the following groups: study centre, gestational age at randomisation (<34 weeks' gestation, 34 to <37 weeks' gestation, ≥37 weeks' gestation), singleton versus twin pregnancy, and highest serum bile acid concentration before randomisation (<40 μmol/L, ≥40 μmol/L). The minimisation algorithm was implemented by a MedSciNet database programmer, with balance and predictability checked by an independent National Perinatal Epidemiology Unit Clinical Trials Unit statistician during the trial. Randomisation was managed via a secure web-based randomisation program.

Ursodeoxycholic acid tablets and placebo tablets were manufactured by Dr Falk Pharma (Freiburg im Breisgau, Germany). The two types of tablet appeared identical in size, shape, and colour. Packs of ursodeoxycholic acid or placebo in identical packaging were produced by the central manufacturing unit at Guy's and St Thomas' Hospital (London, UK) and shipped to site pharmacies. The tablets were packaged for oral administration and did not require any special storage conditions. Packs were labelled with unique pack identifiers according to a randomly generated sequence known only to the manufacturing unit and the trial programmers. A research team member entered baseline data on a web-based database at study enrolment and then allocated a pack number using the web-based randomisation program, which corresponded to a pack for dispensing by that site's pharmacy. If more packs were required, the randomisation program was used to allocate further packs containing the same allocation. Women who were taking ursodeoxycholic acid at enrolment agreed to stop the medication at randomisation.

Trial participants, clinical care providers, outcome assessors, and data analysts were all masked to allocation.

### Procedures

Each film-coated ursodeoxycholic acid tablet contained 500 mg ursodeoxycholic acid (active ingredient) and the following inactive ingredients: magnesium stearate, polysorbate 80, povidone K 25, microcrystalline cellulose, colloidal anhydrous silica, crospovidone, and talc. The placebo tablet contained identical inactive ingredients.

We recommended that women were started on a dose of two oral tablets a day (equivalent to 500 mg ursodeoxycholic acid twice a day in the ursodeoxycholic acid group). The dose was increased by a health-care professional in increments of one tablet a day every 3–14 days if there was no biochemical or symptomatic improvement, to a maximum of four tablets per day (equivalent to 2000 mg ursodeoxycholic acid per day). The dose could be reduced to one tablet a day (equivalent to 500 mg ursodeoxycholic acid a day) at a clinician's discretion (eg, if a woman's bodyweight was <50 kg or if gastrointestinal side-effects occurred). We advised that doses should be spread evenly throughout the day, but that no specific instructions to take with or without food needed to be given. We recommended that treatment should be continued from enrolment until the infant's birth.

Clinical teams reviewed participants at routine-care clinic visits until delivery. Antenatal care, particularly the timing and mode of delivery, was at the discretion of the responsible clinician. Outcomes were recorded on the web-based trial database through case-note review by trained researchers after discharge of the woman and infant.

### Outcomes

The primary outcome was prespecified as a composite of perinatal death (defined as in-utero fetal death after randomisation or known neonatal death up to 7 days after birth), preterm delivery (<37 weeks' gestation), or neonatal unit admission for at least 4 h (from birth until hospital discharge). Each infant was counted once within this composite.

Secondary maternal outcomes, measured at clinical visits between randomisation and delivery, were maternal serum concentrations of bile acids, alanine transaminase (or aspartate transaminase), total bilirubin, and γ-glutamyl transferase, and maternal itch score (measured as the worst episode of itch over the past 24 h in mm on a 100-mm visual analogue scale). Additional secondary maternal outcomes, assessed on case-note review after maternal discharge, were gestational diabetes, mode of onset of labour, and estimated blood loss after delivery.

Secondary perinatal outcomes, assessed on case-note review after infant discharge, included the components of the primary outcome, mode of delivery, birthweight, birthweight percentile, gestational age at delivery, presence of meconium, Apgar score at 5 min, umbilical arterial pH at birth, and number of nights in the neonatal unit. All other secondary outcomes were descriptive only.

Health-resource use post-enrolment was collected at case-note review after maternal and infant discharge. Maternal use was assessed as: total number of nights in hospital (antenatal, intrapartum, and postnatal) together with the level of care, including adult intensive care unit (ICU); mode of delivery; and cost of ursodeoxycholic acid (in intervention group). Infant use was assessed as: total number of nights in the neonatal unit, together with level of care (eg, ICU). Information on outpatient appointments was not collected, because the same outpatient clinical follow-up before the birth was required for both groups.

Research teams undertook standard assessments of safety, with reporting of adverse events and serious adverse events following usual governance procedures for a clinical trial of an investigational medicinal product overseen by the UK Medicines and Healthcare Regulatory Agency.

### Statistical analysis

The sample size was informed by the Cochrane meta-analysis,^9^ in which the event rate for the primary outcome for the infants of untreated women was estimated to be 40%. We calculated that 550 infants of women with intrahepatic cholestasis of pregnancy (275 per group) were required for a 90% chance of detecting (significant at the two-sided 5% level) a reduction in the primary outcome measure from 40% in the control group to 27% in the treated group, corresponding to an absolute risk reduction of 13% and a risk ratio (RR) of 0·675. This estimate was conservative compared with the effect sizes in the Cochrane meta-analysis^9^ for the three individual endpoints (RR 0·31 for perinatal death, 0·46 for preterm delivery, and 0·48 for neonatal unit admission). We planned to recruit 580 women in total, to allow for the possibility of 5% of infants being lost to follow-up. During the recruitment phase, we amended the protocol to permit continued recruitment of additional participants, to allow for women who discontinued the intervention or withdrew from the trial. Interim analyses were done only for presentation to the Data Monitoring Committee, to be reviewed when the committee met at least annually.

The analysis and presentation of results follows the recommendations of the CONSORT group. Full details of the statistical analysis are in the appendix (pp 3–25) and were prespecified.^12^ Statistical analysis was done in Stata version 15. Unmasked data were made available for analysis only after a full database lock (after all data entry had been completed and queries resolved) or on request by the Data Monitoring Committee. All analyses followed the intention-to-treat principle: all randomly allocated women (and infants) were analysed according to the group they were allocated to, irrespective of the treatment they received, if any.

Demographic and clinical data were presented as n (%) for categorical variables, mean (SD) for normally distributed continuous variables, and median (IQR) or median (range) for other continuous variables. All comparative analyses were done adjusting for minimisation factors at randomisation,^13^ with centre as a random effect and the other variables fitted as fixed effects. In addition, for perinatal outcomes for which the denominator was the number of infants, the correlation among twins was accounted for by nesting the mother's identification number as a random effect within centre. Both unadjusted and adjusted effect estimates are presented, but the primary inference is based on the adjusted estimates.

Binary outcomes were analysed using mixed-effect Poisson regression models with robust variance estimation and results were presented as adjusted RRs with 95% CIs.^14^ Continuous outcomes were analysed using mixed-effect linear regression models and presented as adjusted mean differences with 95% CIs. Skewed continuous variables were analysed using quantile regression with minimisation factors (excluding centre) fitted as fixed effects, and results were presented as median differences with 95% CIs. Analysis of outcomes that were measured repeatedly over time (severity of itch and biochemistry measures) used repeated measures models, with means or geometric means of the post-randomisation observations reported,^15^ and the trial groups compared using a mean difference or geometric mean ratio, adjusted for the baseline measures (such that the summary statistics are adjusted for chance imbalances at baseline) and minimisation factors.

Prespecified subgroup analyses were done for the primary outcome and its components, bile acid and itch outcomes, using a statistical test of interaction. Binary outcomes are presented as RRs with 95% CIs on a forest plot. Prespecified subgroups were based on the criteria selected for minimisation: highest serum bile acid concentration before randomisation (<40 μmol/L, ≥40 μmol/L); gestational age at randomisation (<34 weeks' gestation, ≥34 weeks' gestation); singleton or twin pregnancy.

After discussion of the results of the prespecified analysis, and taking into account the findings of a 2019 meta-analysis,^1^ the Trial Steering Committee and Data Monitoring Committee requested two additional post-hoc analyses: the number and percentage of women with peak bile acid of less than 100 μmol/L or at least 100 μmol/L before randomisation, with the primary outcome and its components stratified by this result; and the number and percentage of infants with a spontaneous preterm birth or an iatrogenic preterm birth, with a subgroup analysis of these infants by the minimisation factors specified for the other subgroup analyses. A Kaplan-Meier plot from randomisation to delivery estimate has been included at the request of a reviewer.

Sensitivity analyses were done for the primary outcome, itch score, and bile acid concentration between randomisation and delivery, excluding women or infants of mothers who did not adhere to the intervention (<90% medication adherence consistently self-reported).

In the context of a fixed health-care budget it is important for health-care payers to have information on the potential cost implications of changes in clinical practice. Therefore, we decided to collect data on mother and infant inpatient care and mode of delivery, which were costed using the National Health Service's national schedule of reference costs (appendix p 26).^16^ The cost of ursodeoxycholic acid (derived from the British National Formulary^17^) was included for women who were randomly allocated to receive the intervention. Descriptive statistics are reported, including mean cost per participant and 95% CIs constructed using bootstrapping. Comparative differences in cost were calculated using linear regression, adjusting for gestational age at randomisation, bile acid concentration, singleton versus twin pregnancy, and centre as a random effect. The analysis is from a health-service payer perspective.

The trial is registered with the ISRCTN registry, number 91918806.

### Role of the funding source

The funder of the study had no role in study design, data collection, data analysis, data interpretation, or writing of the report. The corresponding author had full access to all the data in the study and had final responsibility for the decision to submit for publication.

## Results

**Figure 1:**
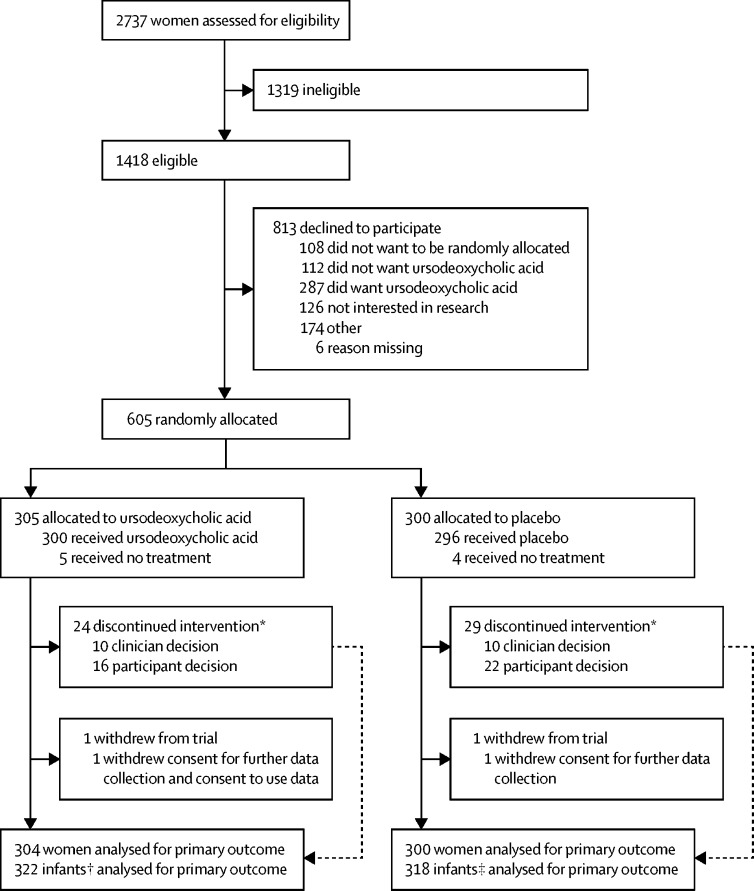
Trial profile *Reasons are not mutually exclusive. †322 of 323 infants born to women allocated to ursodeoxycholic acid; one infant was excluded because the mother withdrew consent for use of data and further data collection. ‡318 of 319 infants born to women allocated to placebo; one infant was excluded because the mother withdrew consent for further data collection.

Between Dec 23, 2015, and Aug 7, 2018, 2737 women were screened, of whom 1418 were found to be eligible (figure 1). We recruited 605 (43%) of 1418 women, including 37 women with a twin pregnancy, across 33 maternity units (appendix p 27). 305 women were randomly allocated to ursodeoxycholic acid, with 304 women and 322 infants included in the primary outcome analysis. 300 women were allocated to placebo, with 300 women and 318 infants included in the primary outcome analysis. Follow-up to maternal and infant discharge continued until December, 2018. Because we met our recruitment target ahead of schedule, we continued recruitment to compensate for the number of women who discontinued the intervention or withdrew from the trial (with approval of the funder, sponsor, and ethics committee), such that our total number of women recruited (n=605) included the target sample size (n=550), the number who discontinued the intervention (n=53) and those who withdrew (n=2). Recruitment ended after 605 women had been enrolled. Baseline characteristics were similar in the two groups (table 1). At enrolment, the groups were well balanced on minimisation factors.

**Table 1:** Maternal baseline characteristics

			**Ursodeoxycholic acid (n=304)**	**Placebo (n=300)**
Age, years			30·5 (5·6)	30·8 (5·3)
Ethnic group				
	White		247 (81%)	246 (82%)
	Black		10 (3%)	7 (2%)
	Asian		34 (11%)	40 (13%)
	Other		11 (4%)	7 (2%)
	Not known		2 (1%)	0
Body-mass index at start of pregnancy (kg/m^2^), mean (SD)			27·4 (6·4)	26·9 (6·1)
Smoked at start of pregnancy			33 (11%)	44 (15%)
Indices of Multiple Deprivation score: quintile 5*, n/N (%)			76/289 (26%)	81/286 (28%)
	Not defined, n		15	14
Previous pregnancy of ≥24 weeks' gestation			178 (59%)	193 (64%)
Previous stillbirth			2 (1%)	2 (1%)
History of intrahepatic cholestasis of pregnancy, n/N (%)			92/175 (53%)	90/190 (47%)
	Missing, n		3	3
Pre-pregnancy liver disease			3 (1%)	6 (2%)
Liver ultrasound at randomisation, n/N (%)			79/293 (27%)	78/292 (27%)
	Normal, n/N (%)		65/77 (84%)	57/77 (74%)
	Gallstones, n/N (%)		9/77 (12%)	12/77 (16%)
	Other abnormality, n/N (%)		3/77 (4%)	8/77 (10%)
	Missing result, n		2	1
Previous operation for gallstones			20 (7%)	17 (6%)
Pre-pregnancy diabetes			4 (1%)	4 (1%)
Gestational age (weeks), median (IQR)†			34·4 (32·1–35·9)	34·4 (31·5–36·0)
	<34 weeks		133 (44%)	131 (44%)
	34 to <37 weeks		141 (46%)	141 (47%)
	≥37 weeks		30 (10%)	28 (9%)
Twin pregnancy†			18 (6%)	19 (6%)
Gestational diabetes			32 (11%)	25 (8%)
Itch score, mean (SD)‡			57·1 (25·1)	59·5 (25·1)
Medication for pruritus§, n/N (%)			146/298 (49%)	137/297 (46%)
	Antihistamine, n/N (%)		121/298 (41%)	119/297 (40%)
	Topical emollient, n/N (%)		102/298 (34%)	101/297 (34%)
	Ursodeoxycholic acid, n/N (%)		15/298 (5%)	13/297 (4%)
	Missing, n		6	3
Highest baseline serum concentration before randomisation				
	Bile acid (μmol/L), geometric mean (95% CI)†		28·1 (26·0–30·3)	26·9 (24·9–29·0)
		<40 μmol/L	232 (76%)	228 (76%)
		≥40 μmol/L	72 (24%)	72 (24%)
	Alanine transaminase, N		286	286
		Alanine transaminase (U/L), geometric mean (95% CI)	70·0 (61·5–79·6)	59·5 (52·0–68·1)
	Aspartate transaminase, N		47	48
		Aspartate transaminase (U/L), geometric mean (95% CI)	49·0 (38·4–62·5)	61·6 (46·8–81·0)
	γ-glutamyl transferase, N		135	138
	γ-glutamyl transferase (U/L), geometric mean (95% CI)		23·3 (20·6–26·4)	21·0 (19·0–23·2)
	Bilirubin, N		289	275
	Bilirubin (μmol/L), geometric mean (95% CI)		8·5 (7·9–9·1)	8·0 (7·4–8·6)

Data are n (%), unless otherwise indicated; N is equal to the total number of participants in the group, unless otherwise indicated; <1% of observations are missing, unless indicated.

* Quintile 5 represents the most deprived geographical areas; scores are not defined for women from Wales.^18^

† Minimisation criterion.

‡ Measured by self-reported worst episode of itch over the past 24 h (mm on visual analogue scale).

§ Not mutually exclusive (some participants used more than one).

The incidence of the primary outcome (perinatal death, preterm delivery, or neonatal unit admission for ≥4 h) did not differ significantly between the groups: 74 (23%) of 322 infants in the ursodeoxycholic acid group versus 85 (27%) of 318 infants in the placebo group had the primary outcome (adjusted RR 0·85 [95% CI 0·62–1·15], p=0·28; table 2). The incidence of the primary outcome components also did not differ significantly between the groups. Three in-utero fetal deaths occurred after randomisation: one in the ursodeoxycholic acid group and two in the placebo group, with two occurring at 35 weeks' gestation and one at 37 weeks' gestation.

**Table 2:** Perinatal outcomes

		**Ursodeoxycholic acid (n=322)**	**Placebo (n=318)**	**Adjusted effect estimate (95% CI)**	**p value**
Perinatal death, preterm delivery,* or neonatal unit admission		74 (23%)	85 (27%)	RR 0·85 (0·62 to 1·15)	0·28
In-utero fetal death		1 (<1%)	2 (1%)	RR 0·51 (0·04 to 6·25)	0·60
Preterm delivery*		54 (17%)	65 (20%)	RR 0·79 (0·57 to 1·10)	0·17
Known neonatal death up to 7 days after birth		0	0	..	..
Neonatal unit admission for ≥4 h		45 (14%)	54 (17%)	RR 0·81 (0·58 to 1·13)	0·21
Livebirth		321 (>99%)	316 (99%)	..	..
Gestational age at delivery, weeks		37·6 (37·1–38·1)	37·4 (37·0–38·1)	Median difference 0·1 (0·0 to 0·3)	0·065
Birthweight, g		3105 (2775–3390)	3040 (2660–3320)	Median difference 94·0 (18·7 to 169·3)	0·014
Birthweight percentile†		59·3 (28·4)	56·3 (27·8)	..	..
	<10th percentile	16 (5%)	18 (6%)	RR 0·89 (0·47 to 1·69)	0·73
	<3rd percentile	7 (2%)	7 (2%)	RR 1·09 (0·38 to 3·12)	0·88
Mode of delivery					
	Spontaneous vaginal (cephalic)	193 (60%)	182 (57%)	RR 1·04 (0·91 to 1·20)	0·56
	Vaginal (breech)	1 (<1%)	3 (1%)	..	..
	Assisted vaginal (cephalic)	21 (7%)	35 (11%)	..	..
	Pre-labour caesarean	71 (22%)	62 (19%)	..	..
	Caesarean	36 (11%)	36 (11%)	RR 1·00 (0·68 to 1·46)	1·0
Presence of meconium-stained amniotic fluid		34 (11%)	52 (16%)	RR 0·65 (0·43 to 0·98)	0·040
Apgar score at 5 min after birth‡		9·0 (9·0–10·0)	9·0 (9·0–10·0)	Median difference 0 (−0·4 to 0·4)	1·0
Apgar score of <7 at 5 min after birth‡, n/N (%)		8/321 (2%)	7/316 (2%)	..	..
Umbilical cord blood sampling, N		112	102	..	..
Umbilical arterial pH		7·2 (0·1)	7·2 (0·1)	Mean difference −0·02 (−0·04 to 0·01)	0·18
Nights in the neonatal unit§		5·5 (3·0–13·0)	6·0 (2·0–16·0)	Median difference 0 (−3·2 to 3·2)	1·0
Main diagnosis for first neonatal unit admission					
	Prematurity, n/N (%)	14/45 (31%)	17/54 (31%)	..	..
	Respiratory disease, n/N (%)	16/45 (36%)	15/54 (28%)	..	..
	Infection suspected or confirmed, n/N (%)	5/45 (11%)	7/54 (13%)	..	..
	Other¶, n/N (%)	10/45 (22%)	15/54 (28%)	..	..

Data are n (%), median (IQR), or mean (SD), unless otherwise indicated; N is equal to the total number of infants in the group, unless otherwise indicated; <1% of observations are missing, unless otherwise indicated. Adjusted effect estimates and p values are shown for primary outcomes, and for secondary outcomes that were prespecified for testing in the published protocol.^11^ RR=risk ratio.

* Delivery at <37 weeks' gestation.

† Calculated using the INTERGROWTH-21st tool.^19^

‡ Data are for livebirths only.

§ Data are for infants with at least one night in a neonatal unit only.

¶ A full list of diagnoses is given in the appendix (p 29).

We found no significant difference in median gestational age at delivery (table 2); time from randomisation to delivery is shown graphically in the appendix (p 28). The proportions of infants who were delivered by spontaneous vaginal birth or caesarean section were similar in the two groups. No significant differences were found between groups in the number of nights spent in the neonatal unit or the main diagnosis for neonatal unit admission (not formally tested). Other perinatal secondary outcomes are shown in table 2 and the appendix (pp 29–30).

**Figure 2:**
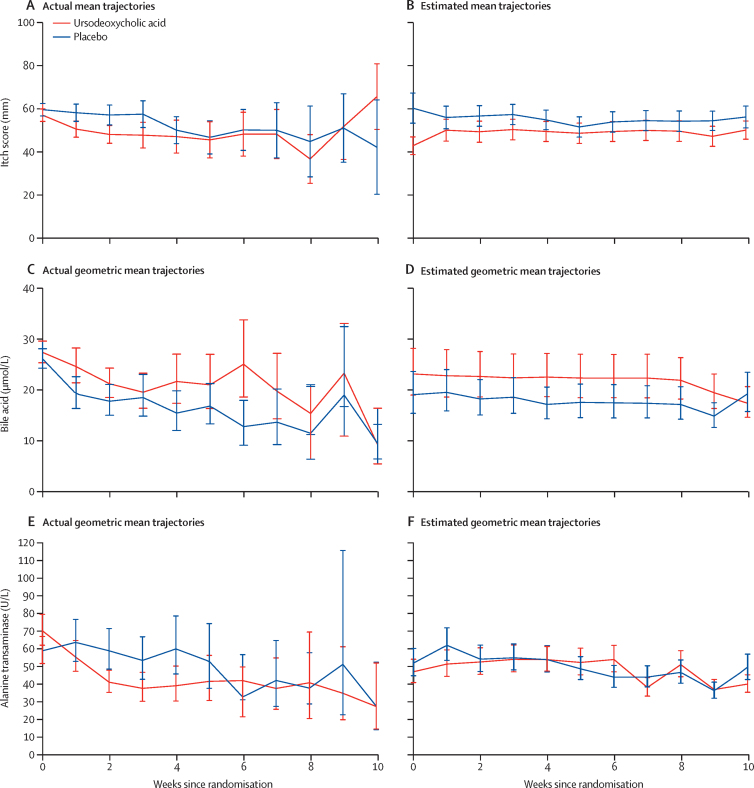
Changes in maternal itch score (A, B), bile acid concentration (C, D), and alanine transaminase concentration (E, F) over 10 weeks post-randomisation Data are actual mean (95% CI) or estimated mean (95% CI). Estimated means are adjusted for baseline measures and minimisation factors.

Post-randomisation maternal itch score was lower in the ursodeoxycholic acid group than in the placebo group (mean difference −5·7 mm [95% CI −9·7 to −1·7], p=0·0054; table 3, figure 2, appendix p 31). Bile acid and alanine transaminase concentrations reduced over time after randomisation in both groups; however, the reduction in bile acid concentration was smaller in the ursodeoxycholic acid group compared with the placebo group (adjusted geometric mean ratio 1·18 [95% CI 1·02 to 1·36], p=0·030). By contrast, a reduction in alanine transaminase concentration was found in the ursodeoxycholic acid group compared with the placebo group (adjusted geometric mean ratio 0·74 (0·66 to 0·83), p<0·0001). Median estimated blood loss was in the normal range for both groups, but was lower in women taking ursodeoxycholic acid than those taking placebo (median difference 50 ml [95% CI −95 to −5]. Other maternal secondary outcomes are shown in table 3 and the appendix (p 32).

**Table 3:** Maternal outcomes

		**Ursodeoxycholic acid (n=304)**	**Placebo (n=300)**	**Adjusted effect estimate (95% CI)**	**p value**
Itch score*, N		241	227	..	..
	Itch score†, mm	49·5 (12·9)	56·9 (13·3)	Mean difference −5·7 (−9·7 to −1·7)	0·0054
Maternal serum bile acid concentration*, N		256	247	..	..
	Maternal serum bile acid concentration† (μmol/L), geometric mean (95% CI)	22·4 (21·4 to 23·5)	18·5 (17·7 to 19·4)	Geometric mean ratio 1·18 (1·02 to 1·36)	0·030
Maternal serum alanine transaminase*, N		242	240	..	..
	Maternal serum alanine transaminase† (U/L), geometric mean (95% CI)	49·5 (43·8 to 55·8)	58·0 (51·0 to 65·9)	Geometric mean ratio 0·74 (0·66 to 0·83)	<0·0001
Gestational diabetes		3 (1%)	9 (3%)	RR 0·33 (0·10 to 1·10)	0·071
Additional therapy for cholestasis†, n/N (%)		134/261 (51%)	125/245 (51%)	..	..
	Antihistamine, n/N (%)	102/134 (76%)	105/125 (84%)	..	..
	Topical emollient, n/N (%)	101/134 (75%)	93/125 (74%)	..	..
	Rifampicin, n/N (%)	1/134 (1%)	2/125 (2%)	..	..
	Open-label ursodeoxycholic acid (tablets stopped), n/N (%)	17/134 (13%)	21/125 (17%)	..	..
	Delivered before first follow-up visit, n	33	42	..	..
	Missing, n	10	13	..	..
Maximum dose of trial medication					
	One tablet once a day	4 (1%)	5 (2%)	..	..
	One tablet twice a day	203 (67%)	198 (66%)	..	..
	One tablet three times a day	62 (20%)	65 (22%)	..	..
	Two tablets twice a day	35 (12%)	32 (11%)	..	..
Mode of onset of labour					
	Spontaneous	33 (11%)	55 (18%)	RR 0·59 (0·42 to 0·83)	0·0025
	Induced or pre-labour rupture of membranes and stimulation	215 (71%)	200 (67%)	RR 1·06 (0·95 to 1·17)	0·30
	Pre-labour caesarean	56 (18%)	44 (15%)	..	..
Initiation of delivery‡					
	Severe maternal symptoms, n/N (%)	17/271 (6%)	28/244 (11%)	..	..
	Maternal serum bile acid, n/N (%)	53/271 (20%)	32/244 (13%)	..	..
	Fetal compromise, n/N (%)	24/271 (9%)	24/244 (10%)	..	..
	Gestational age, n/N (%)	161/271 (59%)	150/244 (61%)	..	..
	Maternal request, n/N (%)	32/271 (12%)	29/244 (12%)	..	..
	Other§, n/N (%)	37/271 (14%)	33/244 (14%)	..	..
Estimated blood loss at delivery, mL		350 (250 to 600)	400 (250 to 600)	Median difference −50 (−95 to −5)	0·029
	<500	195 (64%)	185 (62%)	..	..
	≥500 and <1000	79 (26%)	80 (27%)	..	..
	≥1000	30 (10%)	34 (11%)	..	..

Data are mean (SD), median (IQR), or n (%), unless otherwise indicated; N is equal to the total number of infants in the group unless otherwise indicated; <1% of observations are missing, unless indicated. RR=risk ratio.

* N represents the number of women with data before randomisation, and at least one measurement post-randomisation, included in the model.

† Between randomisation and delivery, adjusted for baseline measures.

‡ Indications are not mutually exclusive (might be more than one indication per participant).

§ Reasons included pre-eclampsia and reduced fetal movement.

Eight serious adverse events were reported, six of which were in the placebo group. None of the serious adverse events were considered to be related to the trial intervention (table 4, appendix p 33). 72 adverse events were reported: 31 in the ursodeoxycholic acid group and 41 in the placebo group. Of note, the same number of patients in each group (n=10) reported adverse events related to gastrointestinal disorders.

**Table 4:** Adverse events and medication discontinuation

			**Ursodeoxycholic acid (n=304)**	**Placebo (n=300)**
Serious adverse events*			2	6
System organ class of serious adverse events				
	Congenital, familial, and genetic disorders		0	1
	Hepatobiliary disorders		0	1
	Infections and infestations		1	1
	Metabolism and nutrition disorders		0	1
	Pregnancy, puerperium, and perinatal conditions		1	1
	Reproductive system and breast disorders		0	1
Adverse events			31	41
	Not related to study intervention		15	31
	Possibly related to study intervention		8	9
	Probably related to study intervention		1	0
	Missing data		7	1
System organ class of adverse events				
	Blood and lymphatic system disorders		4	4
	Gastrointestinal disorders		10	10
	Pregnancy, puerperium, and perinatal conditions		7	18
	Other		10	9
Discontinued intervention			24/304 (8%)	29/300 (10%)
	Clinician decision		10/24 (42%)	10/29 (34%)
		Consultant wanted participant to receive ursodeoxycholic acid	3/10 (30%)	2/10 (20%)
		Increased bile acids or alanine transaminase, or itch, or both	6/10 (60%)	8/10 (80%)
		Nausea, vomiting, or upset stomach	1/10 (10%)	0
	Participant decision		16/24 (67%)	22/29 (76%)
		Itch improved or manageable without medication	1/13 (8%)	1/19 (5%)
		Did not want medication or did not collect medication	5/13 (39%)	4/19 (21%)
		Increased bile acids or alanine transaminase, or itch, or both	6/13 (46%)	8/19 (42%)
		Nausea, vomiting, or upset stomach	1/13 (8%)	2/19 (11%)
		Stopped trial drug for a week	0	1/19 (5%)
		Wanted ursodeoxycholic acid	0	3/19 (16%)
		Not known	3	3
Action after discontinuation				
	Prescribed ursodeoxycholic acid		17/23 (74%)	21/27 (78%)
	Not prescribed ursodeoxycholic acid		6/23 (26%)	6/27 (22%)
	Not known		1	2

Data are number of events or n/N (%). Adverse events and serious adverse events were classified according to system organ class terminology.^20^

* None of the serious adverse events were related to the study intervention.

Around two thirds of women in both groups (203 [67%] of 304 in the ursodeoxycholic acid group and 198 [66%] of 300 in the placebo group) took a maximum of one tablet twice a day (equivalent to 1000 mg ursodeoxycholic acid in the ursodeoxycholic acid group). Similar numbers of women in both groups discontinued the intervention: 24 (8%) of 304 in the ursodeoxycholic acid group and 29 (10%) of 300 in the placebo group, with similar numbers of discontinuations in both groups instigated by clinicians and participants (table 4). In a planned sensitivity analysis, excluding infants whose mothers took less than 90% of the trial medication, a similar proportion of infants had the primary outcome of perinatal death, preterm delivery, or neonatal unit admission for at least 4 h (appendix p 34): 49 (23%) of 217 infants in the ursodeoxycholic acid group compared with 44 (23%) of 190 infants in the placebo group (adjusted RR 0·91 [95% CI 0·63–1·32], p=0·63).

**Figure 3:**
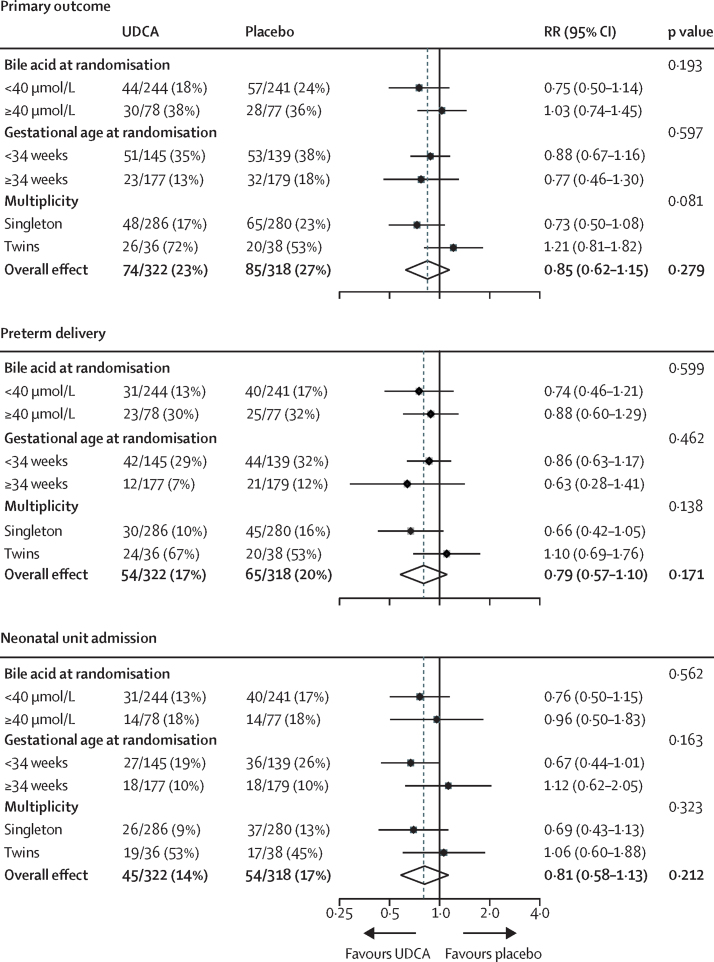
Subgroup analyses for the primary outcome and its main components UDCA=ursodeoxycholic acid. RR=risk ratio.

In planned subgroup analyses (figure 3, appendix p 35), we found no significant interaction of highest bile acid concentration before randomisation (<40 μmol/L, ≥40 μmol/L), gestational age at randomisation (<34 weeks' gestation, ≥34 weeks' gestation), or pregnancy (singleton, twin) with the incidence of the primary outcome, the primary outcome components, itch score, or bile acid concentration post-randomisation.

In requested post-hoc exploratory analyses, the proportion of infants with the primary outcome, whose mothers had a highest bile acid concentration of at least 100 μmol/L at randomisation, was similar in the two groups: nine (39%) of 23 infants in the ursodeoxycholic acid group compared with seven (41%) of 17 in the placebo group (appendix p 32). No difference was found in the proportion of women with spontaneous or iatrogenic preterm birth between the groups (appendix p 36).

We found no significant difference in total cost (maternal costs, infant costs, and cost of ursodeoxycholic acid) between the two trial groups: mean £5420 (SE 284) in the ursodeoxycholic acid group versus mean £5892 (SE 353) in the placebo group (adjusted difference −£429 [95% CI −1235 to 377], adjusted p=0·30; appendix p 37).

## Discussion

In this trial of women with intrahepatic cholestasis of pregnancy, ursodeoxycholic acid was not effective in reducing a composite of adverse perinatal outcomes. Although ursodeoxycholic acid appeared to be safe, it had no clinically meaningful effect on maternal itch symptoms. It did not reduce maternal bile acid concentrations, and the reduction in alanine transaminase was of uncertain clinical significance, given that alanine transaminase is not known to be associated with the risk of stillbirth or preterm labour in intrahepatic cholestasis of pregnancy.^1^ The analysis in women who reported adherence to the intervention reduced the effect size for the primary outcome, and subgroup analyses did not identify any subgroup that showed a greater response to ursodeoxycholic acid. A 2019 meta-analysis^1^ identified that the risk of stillbirth increases in women with intrahepatic cholestasis of pregnancy with peak bile acid concentrations of at least 100 μmol/L, and that the risk of preterm birth increases with peak bile acid concentrations of at least 40 μmol/L. In subgroups of women identified by higher peak bile acid concentration at study enrolment in our study, there was no discernible effect of ursodeoxycholic acid on the primary perinatal composite outcome, its components, or key maternal outcomes. A biologically plausible and clinically important reduction is unlikely to have been missed. We have previously reported that clinicians and patients considered at least a 30 mm (95% CI 15–50) improvement in itch score (from a baseline score of 60 mm) to be a clinically important difference;^10^ therefore, the 5 mm reduction in itch score reported in this trial is unlikely to be clinically useful. Although some secondary outcomes appear to be significantly different (at 95% CIs), the effects do not support a unified action related to ursodeoxycholic acid.

To evaluate real-world effectiveness of the intervention, the trial included pregnant women presenting with pruritus and abnormal maternal bile acid concentrations, which are the criteria commonly used to identify women with intrahepatic cholestasis of pregnancy. Clinicians would be likely to offer ursodeoxycholic acid to these women in the absence of other diagnoses. The study population had a similar range of elevated bile acid concentrations to other multicentre cohorts,^21^ with around three-quarters of women with bile acid concentrations of lower than 40 μmol/L.

A strength of this study was its size, which was considerably larger than any previous trial identified in the literature. The trial was rigorously conducted to a prespecified protocol without changes. The study was done in 33 maternity units in England and Wales, and it included women who were representative of the wider population of pregnant women in terms of demographics and spectrum of disease. Recruitment reached the target number of participants within the prespecified time period, indicating equipoise and a willingness to participate from clinicians and pregnant women.

A limitation of this study was that the incidence of the primary outcome in the control group was lower than that estimated for the sample size calculation. At the time of trial inception, we used the best available data (from the PITCH pilot study,^10^ which had a similar population of women with intrahepatic cholestasis of pregnancy, and the Cochrane systematic review in this field^9^) to estimate the incidence of perinatal death, preterm birth, and neonatal unit admission. In our subsequent individual patient data analysis,^1^ we reported that 412 (13%) of 3080 women with intrahepatic cholestasis of pregnancy had spontaneous preterm birth, which was a much lower incidence than that reported in the Cochrane systematic review (39 [44%] of 89), highlighting the limitations of using small samples to estimate our primary event rate for sample size calculation. The trial primary outcome event rate was reviewed by the Data Monitoring Committee, but because the event rate was similar in the two groups, extending the trial was not considered to be necessary. Although the trial could theoretically have insufficient power to show a difference between groups, the consistent absence of effect in both the analysis of women who adhered to the intervention, and in subgroup analyses of women at greatest risk of adverse perinatal outcomes (bile acid concentration ≥40 μmol/L at enrolment), suggest that this is unlikely.

In contrast to previous meta-analyses,^8^, ^9^ we did not find that ursodeoxycholic acid reduced maternal bile acid concentrations. Enzymic assays used to quantify total serum bile acids detect synthetic ursodeoxycholic acid as well as cholic acid and chenodeoxycholic acid, the pathologically elevated bile acids in intrahepatic cholestasis of pregnancy that are implicated in the pathogenesis of fetal complications.^22^ Treatment with ursodeoxycholic acid reduces the proportion of cholic acid and chenodeoxycholic acid,^22^ but it might be associated with an increase in total bile acid concentration, because of elevated serum concentrations of the drug. Therefore, the interpretation of bile acid concentrations in clinical practice and in this trial is complex. Because we found that bile acid concentrations were reduced even in the placebo group, some women who were initially diagnosed with intrahepatic cholestasis of pregnancy might have had a different disease, with transient raised maternal bile acid concentrations and a different underlying pathophysiology. Other trials in intrahepatic cholestasis of pregnancy have reported a similar reduction in bile acid^10^, ^23^ or alanine transaminase^24^ concentrations in the placebo group. These results highlight that placebo groups might provide the most unbiased assessment of the natural history of a condition, avoiding the selection bias that might occur during enrolment into case series, particularly in treated women.

We considered possible sources of bias for this trial. Selection bias was unlikely because of the randomisation process, including robust allocation-sequence concealment. Performance bias was reduced by effective masking of the intervention to clinicians, patients, and data collectors, such that identification of the active treatment was minimal, with the two groups receiving the same antenatal and intrapartum care pathways. Assessment of the outcome was also masked, minimising detection bias. Differences in attrition between the groups were small, with similar numbers discontinuing the intervention. We aimed to avoid reporting bias by presenting all prespecified secondary outcomes, including the secondary analyses for which effect size measures were calculated, and interpreting secondary outcomes with caution to avoid overinterpretation.

The trial results support the findings of the pilot study,^10^ which reported only a small reduction in maternal itch symptoms, less than that judged by women and health-care professionals to be clinically useful. We have updated the Cochrane systematic review, including data from this trial and four others for stillbirth as an outcome,^10^, ^24^, ^25^, ^26^ two additional trials for spontaneous preterm birth^24^, ^25^ and total preterm birth,^10^, ^23^ and one additional trial for neonatal unit admission.^10^ We confirmed that the pooled results do not show a significant difference in most adverse perinatal outcomes between treatment with ursodeoxycholic acid and placebo. Our meta-analysis showed a significant reduction in total preterm birth, but not spontaneous preterm birth, with ursodeoxycholic acid, probably reflecting a difference in iatrogenic deliveries. In the absence of any discernible effect of ursodeoxycholic acid on iatrogenic preterm birth in women with peak bile acid concentrations at enrolment of more than 40 μmol/L, or in those presenting before 34 weeks' gestation, a strong biological effect of ursodeoxycholic acid seems unlikely.

Ursodeoxycholic acid is the only treatment consistently proposed in guidelines as a disease-modifying drug; no other treatments are widely used for prevention of the adverse perinatal outcomes associated with intrahepatic cholestasis of pregnancy, although a study is planned to evaluate the use of rifampicin.^27^ Our individual patient data analysis^1^ suggests that in women with peak bile acid concentrations lower than 100 μmol/L, the risk of stillbirth is similar to that of the general pregnant population, whereas the risk is significantly higher in women with peak bile acid concentrations of at least 100 μmol/L at any time in the pregnancy. Coexisting pregnancy complications, such as pre-eclampsia or gestational diabetes, might increase the risk of stillbirth.^28^ The only intervention to affect adverse perinatal outcomes is likely to be appropriately planned delivery.

Some subgroups of women with intrahepatic cholestasis of pregnancy, which have not yet been identified, could respond to ursodeoxycholic acid (eg, those with or without comorbidities), either by reduction of maternal symptoms or reduction of adverse perinatal outcomes. For instance, abnormally high bile acid concentrations are associated with stillbirth in intrahepatic cholestasis of pregnancy,^1^ and in-vitro studies have shown that ursodeoxycholic acid might be protective against bile-acid induced cardiac arrhythmias,^29^ potentially mediated through reduction of specific bile acid species.^22^ However, further understanding of the pathophysiology underpinning stillbirth (and therefore the target for intervention) is needed. Additional work is also needed to confirm the likely pruritogen in intrahepatic cholestasis of pregnancy to identify a target for therapeutic treatments; progesterone sulfates^30^ and lysophosphatidic acid^31^ have been proposed as candidates. However, the lack of in-vivo evidence of benefit should preclude further routine clinical use of ursodeoxycholic acid, even in the absence of harm, to avoid women being offered an unproven treatment.

## Data sharing

The dataset will be available to appropriate academic parties on request from the Chief Investigator, Lucy Chappell, in accordance with the data sharing policies of King's College London and the National Perinatal Epidemiology Unit Clinical Trials Unit, with input from the co-investigator group where applicable, subject to submission of a suitable study protocol and analysis plan, on publication of all initial trial results.
